# Molecular Biomarkers in Bladder Cancer: Novel Potential Indicators of Prognosis and Treatment Outcomes

**DOI:** 10.1155/2016/8205836

**Published:** 2016-01-26

**Authors:** Masayoshi Nagata, Satoru Muto, Shigeo Horie

**Affiliations:** ^1^Department of Urology, Graduate School of Medicine, Juntendo University, 3-1-3 Hongo, Bunkyo-ku, Tokyo 113-8431, Japan; ^2^Department of Urology, School of Medicine, Teikyo University, 2-11-1 Kaga, Itabashi-ku, Tokyo 117-003, Japan

## Abstract

Although many clinical and molecular markers for predicting outcomes in bladder cancer (BC) have been reported, their application in clinical practice remains unclear. Bladder carcinogenesis has two distinct molecular pathways that direct the development of BC.* FGFR3* mutations are common in low-grade BC, while* TP53* mutation or loss of* RB1* is associated with muscle-invasive BC. However, no tissue-based gene markers confirmed by prospective large-scale trials in BC have been used in clinical practice. Micro-RNA analyses of BC tissue revealed that miR-145 and miR-29c^⁎^ function as tumor suppressors, whereas miR-183 and miR-17-5p function as oncogenic miRNAs. In liquid biopsy, circulating tumor cells (CTC), exosomes, or cell-free RNA is extracted from the peripheral blood samples of cancer patients to analyze cancer prognosis. It was reported that detection of CTC was associated with poor prognostic factors. However, application of liquid biopsy in BC treatment is yet to be explored. Although several cell-free RNAs, such as miR-497 in plasma or miR-214 in urine, could be promising novel circulating biomarkers, they are used only for diagnosing BC as the case that now stands. Here, we discuss the application of novel biomarkers in evaluating and measuring BC outcomes.

## 1. Introduction

Bladder cancer is the most common cancer of the urinary tract, with an incidence rate of 350,000~380,000 cases being reported per year worldwide [1, 2]. Its main histological type is urothelial carcinoma. There are two clinical phenotypes of bladder cancer: (1) non-muscle- invasive bladder cancer and (2) muscle-invasive bladder cancer. Non-muscle-invasive cancers frequently recur at approximate rate of 50~70% and progress to muscle-invasive disease at rate of 1~2% and ~45% in low-grade and high-grade tumors, respectively [3–5]. Muscle-invasive cancers have a 5-year survival rate of <50% [6, 7]. As it stands now, treatment for muscle-invasive bladder cancer is chiefly total cystectomy. Therapeutic methods against advanced bladder cancer are chemotherapies that mainly use cisplatin as the first-line treatment and have still not made significant progress for more than 20 years.

A set of clinical and pathological parameters are used for risk stratification in bladder cancer, such as number of tumors, size of the tumor, prior recurrence rate, T-stage, presence of carcinoma* in situ*, tumor grade, lymph node status, and variant histology. However, they are only limited to predicting clinical outcomes [8]. Novel therapies for advanced muscle-invasive bladder cancer and established predictive biomarkers on response to them are yet to be identified. Hence, predictive biomarkers for the development of target therapy and novel therapies for advanced bladder cancer must be identified.

Here, we discuss the application of molecular predictive biomarkers in advanced muscle-invasive bladder cancer as well as molecular markers of poor prognosis in postcystectomy patients. Moreover, we discuss the current findings of liquid biopsy in patients with advanced bladder cancer as well as those of histopathological analyses of primary bladder cancer.

## 2. Gene Abnormalities in Primary Bladder Cancer

Some theories on the putative molecular mechanism underlying bladder cancer have been demonstrated. Firstly, it is important to understand the molecular pathway of carcinogenesis in bladder cancer before exploring prognostic and predictive molecular markers of advanced bladder cancer. The concept of “field cancerization” was introduced in 1953, which is the theory of multicentric cancer origins [9]. A population of cells in morphologically normal epithelium possessed common genetic or epigenetic aberrations, similar to that observed in bladder cancer, which might provide a ground for multiple tumorigenesis [10]. On the other hand, the “clonal origin” theory states that bladder tumors arise from the uncontrolled spread of a single transformed cell that can grow independently with variable subsequent genetic alterations [11]. Since recent molecular biological approach showed various samples from metachronous and synchro-allopatric tumors could arise from monoclonal origin by analyses according to the pattern of X-chromosome inactivation,* TP53* mutation, and loss of heterozygosity, “clonal origin” theory is currently supported on bladder cancer [11–13]. Then, a family of genes has been characterized that follows this “two-hit” model including the two prototype suppressor genes: the retinoblastoma 1 (*RB1*) and* TP53* genes [14]. It is now well established that accumulation of genetic alterations forms the basis for progression from a normal cell to a cancer cell, referred to as the process of multistep carcinogenesis [15, 16]. Recent analyses demonstrated that non-muscle-invasive and muscle-invasive bladder cancers have distinct pathways in carcinogenesis. One pathway involves mutation of* FGF receptor 3*, thereby giving rise to low-grade non-muscle-invasive papillary tumors that frequently recur but seldom invade. In contrast, muscle-invasive bladder cancer and carcinoma* in situ* exhibit deletions or mutations of the* TP53*,* RB1*,* ERBB2*, or* PTEN* [16].

Originally, the* HRAS* gene was the first human oncogene identified from a human bladder cancer cell line. A point mutation alters the 12th amino acid of the* HRAS* oncogene product p21 [17]. It was reported that HRAS was frequently overexpressed in non-muscle-invasive cancer [18]. However, recent reports showed that the* RAS* genes mutations are present in only 1–13% of bladder cancer and less frequent in muscle-invasive cancer [19–21]. Moreover, the* RAS* gene mutation was not a predictor for disease-specific survival [22].

FGFR3 (fibroblast growth factor receptor 3), a receptor tyrosine kinase, is one of the most frequently mutated genes in bladder cancer. The rate of mutation in non-muscle-invasive bladder cancer is 60–70% [23–25]. FGFR3 plays a critical role in bladder cancer from low-grade stage [26], which is characterized by low levels of protein synthesis and high cell cycle gene activity [27]. However, its mutation is comparatively less common in patients with muscle-invasive bladder cancer at the time of diagnosis and has not been established to be as a prognostic biomarker in advanced bladder cancer.

TP53, a transcription factor, has many functions, such as induction of apoptosis, inhibition of cell proliferation, and arrest of the cell cycle. Nuclear accumulation of TP53 is a predicting factor of poor prognosis in advanced bladder cancer [28, 29]. In multivariable analyses of 692 patients with invasive cancer treated with radical cystectomy and lymphadenectomy, TP53 expression was independently associated with disease recurrence and cancer-specific mortality [30]. However, the authors mentioned that assessing TP53 expression has limited utility in patients with lymph node-positive bladder cancer. In addition, the* TP53* gene alteration, which is a poor prognostic factor, is found in 53% of patients who underwent cystectomy [31].

The* RB1*, a tumor suppressor gene, is a negative regulator of the cell cycle, and its alterations are related to carcinogenesis in several cancers. Loss of RB1 expression is an adverse prognostic biomarker in muscle-invasive bladder cancer [32]. Bladder cancer with mutation of the* RB1* gene exhibits low FGFR3 levels and is associated with significantly poor disease-specific survival [33]. The* TSC1* is a tumor suppressor gene, located on chromosome 9q34. TSC1 is a negative regulator of mTOR signaling in complex with TSC2. Deletions of the long arm of chromosome 9 are the most common genetic alteration in bladder cancer. TSC1, and TSC2, which constitute the mTOR regulatory tuberous sclerosis complex, are mutated at a combined frequency of 15% [34]. Thus, everolimus might have activity in metastatic urothelial cancer patients who harbor the* TSC1* mutation. The* TSC1* mutation possibly has a causative role in the progression and this process is possibly related to the functional loss of p27 [35].

Other genes, such as the* ERBB2/HER2* [36, 37] and* PTEN* [38], have been reported to be involved in the progression of advanced bladder cancer. Thus, the signaling pathway from receptors of tyrosine kinase to AKT/PI3K would definitely play a role in carcinogenesis and development of bladder cancer and other cancers. However, only analysis of promising single gene predictive biomarker in bladder cancer has limitations to apply to clinical practice. Further prospective evaluation would be required.

## 3. MicroRNA Expression in Primary Bladder Cancer

MicroRNAs (miRNAs) are 18–24-nucleotide-long noncoding RNA that inhibit gene function by endogenous blocking. Several miRNAs are involved in carcinogenesis as tumor suppressor or oncogenic molecules.

miR-145, miR-143, and miR-125b are tumor suppressors that are downregulated in bladder cancer tissue, whereas miR-183, miR-96, miR-17-5p, and miR-20a are oncogenic miRNAs that are upregulated in bladder cancer tissue [39]. miR-145 is one of the most recurrently downregulated miRNAs in bladder cancer. miR-141 and miR-205 are poor prognostic biomarkers of overall survival in bladder cancer [40]. miR-29c^*∗*^ expression is severely decreased in advanced cancer. Non-muscle-invasive bladder cancer (50%) with low miR-29c^*∗*^ expression subsequently progressed, whereas 94% of non-muscle-invasive bladder cancers with high expression did not progress [41].

## 4. “Liquid Biopsy” with Patients' Blood Samples: Circulating Tumor Cells, Exosomes, and miRNAs (Figure 1)

Advanced technology uses patients' blood or urine as samples instead of primary bladder cancer tissue to analyze bladder cancer prognosis and to explore novel prognostic or predictive biomarkers. Liquid biopsy involves the analyses of circulating tumor cells (CTCs), exosomes, and circulating miRNAs in patients' blood or urine in human cancers [42]. In the CORRECT analysis for advanced colorectal cancer, a high concordance was observed between plasma circulating DNA and tumor tissue for the* KRAS* and* PIK3CA* mutations [43]. We have been able to select the most effective drug to treat patients with advanced prostate cancer by the CTC assay, using patients' peripheral blood samples. Detection of AR-V7 (Androgen-Receptor splice Variant 7 messenger RNA) in CTCs from patients with castration-resistant prostate cancer may be associated with resistance to enzalutamide and abiraterone [44].

The CTC assay has a potential role in the management of bladder cancer. Next-generation sequencing analysis showed somatic variant detection in 50% of patients with neoadjuvant bladder cancer [45]. At an early stage of diagnosis for bladder cancer, quantitation of CTC from blood and urine by FR*α* (folate receptor *α*) ligand-targeted polymerase chain reaction (PCR) could be a promising method for diagnosis [46]. In addition, it was reported that detection of CTC was associated with poor prognostic factors. CTCs were detected in 20% of patients with high-risk non-muscle-invasive bladder cancer and effectively predicted both time-to-recurrence and progression-free survival [47]. Another study showed that the presence of CTC in patients with metastatic bladder cancer was associated with poor survival. However, there was no difference in survival between the CTC-positive and CTC-negative patients with localized bladder cancer [48]. Preoperative CTCs in peripheral blood are detected in 23% of non-metastatic advanced bladder cancer. There was concordance between HER2 expression on CTC and the* HER2* gene amplification status of the primary tumor and lymph node metastases in CTC-positive cases [49]. Their studies had limitations in the small sample size.

Exosomes are small (30–100 nm) membrane vesicles released into the extracellular environment on fusion of multivesicular bodies with the plasma membrane [50]. Exosomes play several physiological roles, such as immune response modulation, presentation of antigens to immune cells, intercellular communication through transfer of proteins, mRNA, and miRNA. However, no* in vivo* research on exosomes has been reported for bladder cancer. Exosomes isolated from the urine of patients with muscle-invasive bladder cancer induced epithelial-to-mesenchymal transition in urothelial cells [51]. A new insight into the role of exosomes in transition of bladder cancer into muscle-invasive cancer was provided. Thus, exosome research in advanced bladder cancer could be a new platform for predicting progression and innovating targeted therapy.

miRNAs are found within cells and in serum and other body fluids. The function of these extracellular circulating miRNAs is not well understood. Extracellular miRNAs embedded in circulating exosomes serve as prognostic biomarkers in cancer. A number of plasma exosomal RNAs are reported as being diagnostic, prognostic, or even therapeutic biomarkers in cancer patients [52]. Circulating exosomal RNAs contain various RNA species and changes in exosomal RNA contents are robust candidates as clinical biomarkers for advanced prostate cancer [53, 54]. Circulating miR-497 and miR-663b in plasma were expressed with significant difference in bladder cancer [55]. Although these miRNAs could be promising novel circulating biomarkers, they are used for only diagnosing bladder cancer as the case now stands. Thus, circulating miRNAs in blood or urine could be biomarkers at several clinical stages, such as detection of bladder cancer, prediction of transition to muscle-invasive disease, or prediction of outcomes. However, urinary cell-free miR-214 or miR-155 has been reported to be used only as a diagnostic biomarker [56, 57], and few studies have reported the relationship between circulating miRNAs and bladder cancer.

In the near future, liquid biopsy detecting CTCs, exosomes, and miRNAs will serve as a tool to predict outcomes and to select effective targeted therapies. Although circulating cells and DNA analyses would be promising because of their convenient and minimally invasive procedures, they are limited by their sample collection methods, lack of sensitivity and specificity, or high costs.

## 5. Conclusions

Despite the impressive development of recent research on CTC, microRNA, and exosomes, our knowledge pertaining to the biology of bladder cancer lags behind that pertaining to other solid cancers. Therapeutic methods against advanced bladder cancer have still not made significant progress for more than 20 years, since chemotherapies mainly use cisplatin as the first-line treatment of choice for advanced bladder cancer. One of the reasons for this could be the dramatically different mechanisms underlying carcinogenesis in non-muscle-invasive bladder cancer and muscle-invasive high-grade cancer, which could complicate molecular research on bladder cancer. Establishment of molecular profiling from the context of large clinical trials is required to stratify patients before treatment with conventional chemotherapy. However, currently available data suggest that single biomarkers are inadequate for the surveillance of high-risk patients. Owing to this new angle, combinations of considerably different approaches, such as the use of epigenetic and genetic biomarkers, primary tissue and CTC samples, and genomics and proteomics, are being proposed for successful validation of a robust prediction tool for bladder cancer. Moreover, novel effective agents with the ability to increase the overall survival rate of bladder cancer need to be identified for clinical use.

## Figures and Tables

**Figure 1:**
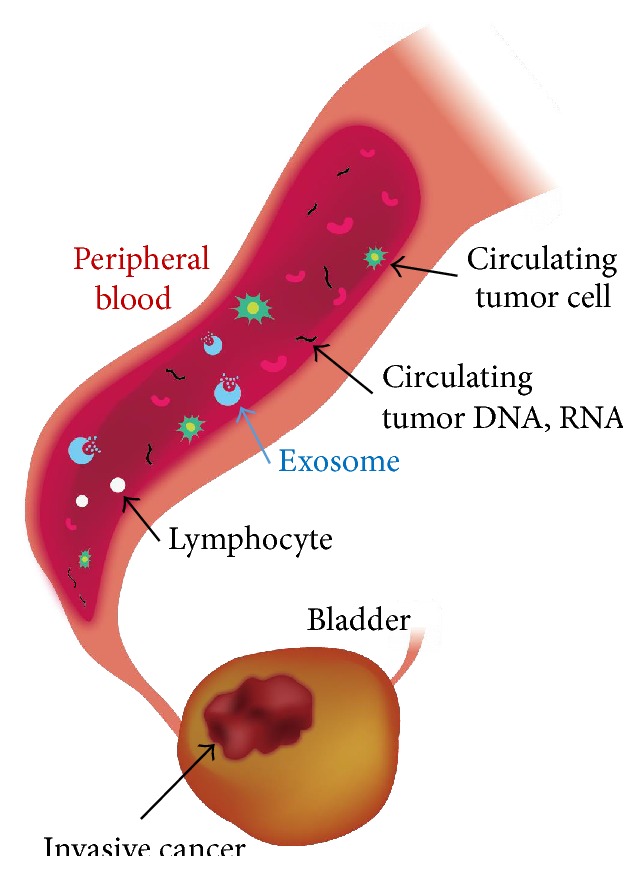
Schema of liquid biopsy.
